# Editorial: Biology-Inspired Engineering and Engineering-Inspired Biology

**DOI:** 10.3389/fnbot.2020.614683

**Published:** 2020-11-13

**Authors:** Jan-Matthias Braun, Poramate Manoonpong, Xiaofeng Xiong

**Affiliations:** ^1^Applied AI & Data Science, The Mærsk Mc-Kinney Møller Institute, University of Southern Denmark, Odense, Denmark; ^2^Embodied AI and Neurorobotics Lab, SDU Biorobotics, The Mærsk Mc-Kinney Møller Institute, University of Southern Denmark, Odense, Denmark; ^3^Bio-Inspired Robotics and Neural Engineering Lab, School of Information Science and Technology, Vidyasirimedhi Institute of Science and Technology, Rayong, Thailand

**Keywords:** biology-inspired engineering, engineering-inspired biology, bio-inspired computation, bio-inspired sensors, bio-inspired materials, bio-inspired structure

The term biology-inspired engineering refers to the fact that biology has been an important inspiration for developments in all aspects of engineering, for example the design of robots. Bio-inspired computation (Manoonpong and Tetzlaff, 2018; Manoonpong et al., 2020), sensors (Steffen et al.), actuators (Vanderborght et al., 2013; Dicker et al., 2017; Lee and Oh, 2019; Lund et al., 2019), structures (Tramsen et al., 2018; Tan et al., 2019; Shao et al., 2020), and materials (Rafsanjani et al., 2018; Wang et al., 2020) have enabled robots to produce robust and comparable behaviors to their biological counterparts. Owing to the biologically comparable behaviors, interdisciplinary biologists tend to flip the approach, i.e., engineering-inspired biology, whereby engineering systems and principles are utilized to initiate and test new hypotheses in biological research. For example, robots have been used as tools to investigate and test animal functions (Ijspeert, 2014; Dürr et al.; Wei et al.). This special issue reports the results and reviews of biology-inspired engineering and engineering-inspired biology research.

## 1. Biology-Inspired Engineering

In Schiller et al., a gecko-inspired soft robot was developed to climb inclined, flat surfaces. By reducing the volume and increasing the self-reinforcing effect of the bending actuators of the robot and using gecko inspired locomotion, they can reduce the energy consumption of the robot, and at the same time, improve the robot climbing ability and the movement speed. Results show that the robot with its straight gait can climb slopes of up to 84 degrees. In the horizontal plane, it can achieve a locomotion speed of 6 cm/s. In Zhu et al., neurobiologically inspired control for the coordinated behavior and coupled locomotion of a multi-joint bionic robot was developed. It is based on central pattern generators (i.e., σ-Hopf oscillators) with an undistorted synchronization method. Results show that the control approach can be used for the locomotion control of a bionic parallel waist of a legged robot, which is a highly coupled system. In principle, the approach can benefit the transition and transformation of the locomotion and makes the coupling motion more flexible; thereby it can be applied to not only legged robots but also flying, and swimming robots, etc. In Dai et al., an affine transformation based on an artificial bee colony algorithm was proposed to greatly improve the positioning precision under large initial errors condition. The affine transformation is applied to the initialization and evolutionary processes of the algorithm. The proposed algorithm provides a novel way to increase the matching accuracy of gravity aided inertial navigation systems. In Sun et al., a new modular, small-sized, bio-inspired quadruped robot called Lilibot was introduced. Its modular components can be flexibly reconfigured to obtain features, such as different leg orientations for investigating the effectiveness and generalization of bio-inspired self-organized locomotion control. It has multiple sensory feedback to support bio-inspired vestibular reflexes and compliant control mechanisms for body posture stabilization and compliant behavior, respectively. Lilibot was evaluated through a bio-inspired adaptive neural controller. Results show that it can autonomously perform various adaptive behaviors including self-organized locomotion under different leg orientations, adaptive body posture stabilization on a tiltable plane, and adaptive leg compliance for unexpected external load negotiation. The results suggest that Lilibot can be considered as a friendly and generic quadrupedal platform for biology-inspired engineering and engineering-inspired biology studies. Specifically, it can be used as a tool to develop bio-inspired control in engineering research and to investigate and test animal functions as well as new hypotheses in biological research.

Going under water, Plum et al. presents a biologically inspired underwater vehicle with a soft and compliant exoskeleton. It is controlled by a shallow neural network, trained by a genetic algorithm. The compliant exoskeleton reduces accelerations induced by collisions dramatically, thus reducing stress on the vehicles components as well as reaction forces on the vehicles surroundings. Employing a segmented structure, the spherical exoskeleton does not limit the use of cameras, sensors, or manipulators. This study shows how a versatile vehicle can achieve protection against severe damage of often fragile underwater surroundings, like coral reefs, or of accompanying humans by means of biologically inspired engineering. It thereby presents a convincing, practical application in a very complex environment, which is achieved by basic changes to the structure, instead of costly changes to components and even more complex control algorithms.

Steffen et al. focuses on biology-inspired vision. For the review, sensors and algorithms for event-based stereo vision were investigated. These biologically plausible vision systems add time as additional constraint to solve the correspondence problem, i.e., for finding matching points in the two images of a stereo vision system. The event based nature of the vision sensors makes them perfect for further processing in spiking neural networks. The review covers conventional electrical as well as biological sensors, depth perception, and thus enables a thorough understanding of how biologically-plausible vision systems were developed, how their data can be processed, how they can improve future applications of vision systems, but also where there are still shortcomings in this very dynamic field of neuromorphic processing of sensory data.

Based on experiments to determine the maximum cooperative grasping mass and diameter of the human thumb and index finger, Chen et al. present non-linear regression models which relate these variables to age, gender, sum of thumb and index finger lengths, the ratio of index finger to thumb, and the hand used. The authors were successfully applying these two models to design the optimal size of robotic hands to reproduce the experiments. With this research, the authors not only demonstrate how biological findings can be used to determine relevant properties of a robotic model, they also argue that their models can be applied to hand rehabilitation. Thereby, they close the loop from biology-inspired engineering back to engineering-inspired biology, showing that both research directions are inherently intertwined.

## 2. Engineering-Inspired Biology

In Kano, Kanauchi, Aonuma et al., the mechanism how brittle stars make decisions about their moving direction in decentralized nervous systems is developed by capturing behavioral findings in a phenomenological mathematical model. The authors propose a decision making process based on self-organization, which can autonomously determine the moving direction and move toward it adaptively. Based on the model, simulations verify that the model is able to reproduce the behavioral observations. This work is extended in Kano, Kanauchi, One et al., where a brittle star-like robot was introduced and exploited as a tool to realize an autonomous control mechanism underlying the locomotion and adaptation of brittle stars. According to this, the decentralized control mechanism can autonomously generate the inter-arm and intra-arm coordination for locomotion and adapt to unexpected physical damages, in one or several arms, by automatically coordinating the still responsive arms, in a way similar to brittle stars. On one hand, the study demonstrated that an engineering-inspired biology approach can be used to reveal the essence of the inter- and intra-arm coordination mechanism in brittle stars. On the other hand, it paves the way to develop robot control that can coordinate a large number of bodily degrees of freedom and adapt to unpredictable circumstances in real-time. In Dürr et al., the six legged robot HECTOR was developed for integrative biomimetics research of hexapedal locomotion. HECTOR has 18 highly sensorized compliant actuators, a light-weight exoskeleton, a distributed and expandable hardware architecture, as well as a proper dynamic simulation framework. These characteristics enable HECTOR to be an appropriate robot for investigating insect locomotion intelligence (e.g., muscle compliance and distributed proprioception). In Wei et al., a bio-inspired robot was built to validate the assumption that the flapping wings and leg burrs of locusts have an influence on their jumping performance. The validation has shown that the leg burrs and flapping wings make the locusts jump longer and improve their landing stability.

## 3. Concluding Remarks

The articles collected in this research topic present an overview of recent developments reflecting the 2-fold interaction between engineering and biology. In biology-inspired engineering, we see how innovative solutions can result from research on their biological counterparts. Approaches span a broad range from mimicking material properties to re-engineering a biological mechanism based on an abstract principle. In engineering-inspired biology, the articles do not only showcase how engineering solutions can demonstrate the level or depth of our understanding of a mechanism, but also how they allow us to validate or reveal possible mechanisms in the biological counterpart. The success of this two-fold interaction makes it likely that in the future we will see more research that transfers not only in one direction, but that shows mutual inspirations and direct interactions between biological research and engineering applications, as for example in healthcare, rehabilitation, search and rescue, inspection, and space exploration.

## Author Contributions

All authors listed have made a substantial, direct and intellectual contribution to the work, and approved it for publication.

## Conflict of Interest

The authors declare that the research was conducted in the absence of any commercial or financial relationships that could be construed as a potential conflict of interest.
